# The impact of passive heat maintenance strategies between an active warm-up and performance: a systematic review and meta-analysis

**DOI:** 10.1186/s13102-022-00546-7

**Published:** 2022-08-13

**Authors:** Gavin Cowper, Stuart Goodall, Kirsty Hicks, Louise Burnie, Marc Briggs

**Affiliations:** Faculty of Health and Life Sciences, Northumbria University, Newcastle upon Tyne, UK

**Keywords:** Clothing, Core temperature, Muscle temperature, Passive heating, Peak power, Performance, Temperature

## Abstract

**Background:**

Prior to exercise, a warm-up routine has been suggested to be an imperative factor in task readiness with the anticipation that it will enhance performance. One of the key benefits of a warm-up is the increase in muscle and core temperature, which can be achieved in a variety of ways. An effective way to achieve improvements in core and muscle temperature is by performing an active warm-up. However, lengthy transition periods between an active warm-up and exercise performance are known to cause a decline in core and muscle temperature, thereby reducing performance capability. As such, methods are needed to assist athletes during transition periods, to maintain the benefits of a warm-up with the aim of optimising performance. Accordingly, the purpose of this review is to systematically analyse the evidence base that has investigated the use of passive heating to aide sporting performance when a transition period is experienced.

**Methods:**

A systematic review and meta-analysis were undertaken following relevant studies being identified using PubMed, Web of Science, and EBSCO. Studies investigating the effects of passive heating strategies during the transition period between an active warm-up and exercise performance were included. The quality of the included studies were assessed by two independent reviewers using a modified version of the Physiotherapy Evidence Database scale.

**Results:**

Seven studies, all high quality (mean = 7.6), reported sufficient data (quality score > 5) on the effects of passive heating strategies on exercise performance, these studies consisted of 85 well-trained athletes (78 male and 7 female). Passive heating strategies used between an active warm-up and exercise, significantly increased peak power output in all studies (ES = 0.54 [95% CI 0.17 to 0.91]). However, only a favourable trend was evident for exercise performance (ES = 1.07 [95% CI − 0.64 to 0.09]).

**Conclusions:**

Based upon a limited number of well-conducted, randomised, controlled trials, it appears that passive heating strategies used between an active warm-up and exercise have a positive impact on peak power output. Although, additional research is necessary to determine the optimum procedure for passive warm-up strategies.

**Supplementary Information:**

The online version contains supplementary material available at 10.1186/s13102-022-00546-7.

## Introduction

Before exercise, a warm-up routine has been suggested to be an imperative factor in task readiness with the anticipation that it will enhance performance [1]. One of the key benefits of a warm-up is the increase in muscle and core temperature and this can be achieved in a variety of ways [2]. The most effective way to achieve improvements in core and muscle temperature is by performing an active warm-up, which involves a series of dynamic movements [3]. The subsequent increase in muscle temperature results in various physiological advantages, including an increased speed of contraction and relaxation of muscle fibres, increased anaerobic metabolic capacity and improved nerve conduction within the peripheral, and central, nervous systems [4]. Therefore, maximising the effectiveness of the active warm-up phase prior to sports performance is an integral part of preparation. After the active warm-up period, athletes can often wait between 10 and 30 min before the start of competition (transition period). Zochowski et al. found that a reduction in the length of the transition period yields performance benefits, whereas a lowering of core and muscle temperature occurs when transition periods are prolonged [5, 6]. However, it can be challenging to alter competition schedules by a substantial margin. As such, methods are needed to assist athletes through transition periods, to maintain elevated core and muscle temperatures gained from a warm-up with the aim of optimising performance.

Initially, hot showers or baths were used to combat the decline in muscle and core temperature in swimming [7, 8], however, logistical, and practical issues make these methods difficult to use in the majority of sporting scenarios. Therefore, recent investigations have developed methods to maintain an elevated muscle and core temperature during the transition phase between the warm-up, and subsequent exercise performance. Faulkner et al. were the first to implement a passive heating device following an active warm-up [9]. Specifically, these authors investigated the use of heated tracksuit pants in the marshalling period before a sprint cycling race, and the intervention significantly reduced the decline of muscle temperature whilst peak power output improved. Additionally, similar passive heating garments and blizzard survival jackets have been used to manipulate body temperature during the transition phase and have improved bobsled [10], rugby [11], swimming [12, 13], and rowing [14] performance.

Thus, passive heating seems to be a logical, practical intervention to maintain warm-up-induced elevations in core and muscle temperature throughout the long transition periods observed in sports competitions. The use of a heat maintenance device during the transition period following an active warm-up, and prior to competition, has the capacity to yield performance enhancing benefits akin to those of an active warm-up. Therefore, the purpose of this review is to systematically analyse the evidence base that has investigated the use of passive heating in the transition period to aide sporting performance.

## Methods

### Search strategy

A systematic review was conducted according to Preferred Reporting Items for Systematic reviews and Meta Analyses guidelines (PRISMA) [15]. Relevant publications published prior to December, 2021 in three databases (PubMed, Web of Science & EBSCO) were identified. The search was performed using the Boolean search criteria, which limited the search results with operators including AND/OR to only those documents containing relevant key terms in the scope of this review (see Table 1).

**Table 1:** Search strategy and inclusion/exclusion criteria based on PICO (population, intervention, comparison and outcome)

Databases	Search terms	PICO	Inclusion criteria	Exclusion criteria
PubMed, EBSCO, Web of Science	((sport* OR exercise* OR perform*) AND (“post-warm-up” OR “warm-up” OR “half-time” OR “rewarming” OR “re-warming” OR “passive warming” OR “interval” OR “quarter”) AND (“passive heat*” OR “heat* jacket” OR “heat* pants” OR “blizzard survival jacket*” OR “heat* garment”))	Population	Trained athletes	Non-trained athletes
		Intervention	An active warm-up combined with a passive heating intervention	No passive heating intervention applied following an active warm up
		Comparison	Passive heating strategies and control	No comparison between a passive heating intervention and control
			Core and/or muscle temperature variations during passive heating intervention	No core and/or muscle temperature variations during passive heating intervention
		Outcome	Performance measures (time to competition, distance covered)	Performance measures not recorded

### Eligibility search

Research articles were included or excluded using criteria defined with the Population, Intervention, Comparison and Outcome criteria (PICO) [16], and the literature searches were limited to studies involving trained athletes, defined as an athlete who regularly competes in key regional or national tournaments in their specific sport. Grey literature such as thesis dissertations, and conference abstracts were excluded. Additionally, all included studies had to be written in the English language and had to be published in a peer-reviewed journal. The search strategy and eligibility criteria are shown in Table 1.

### Study selection

**Fig. 1:**
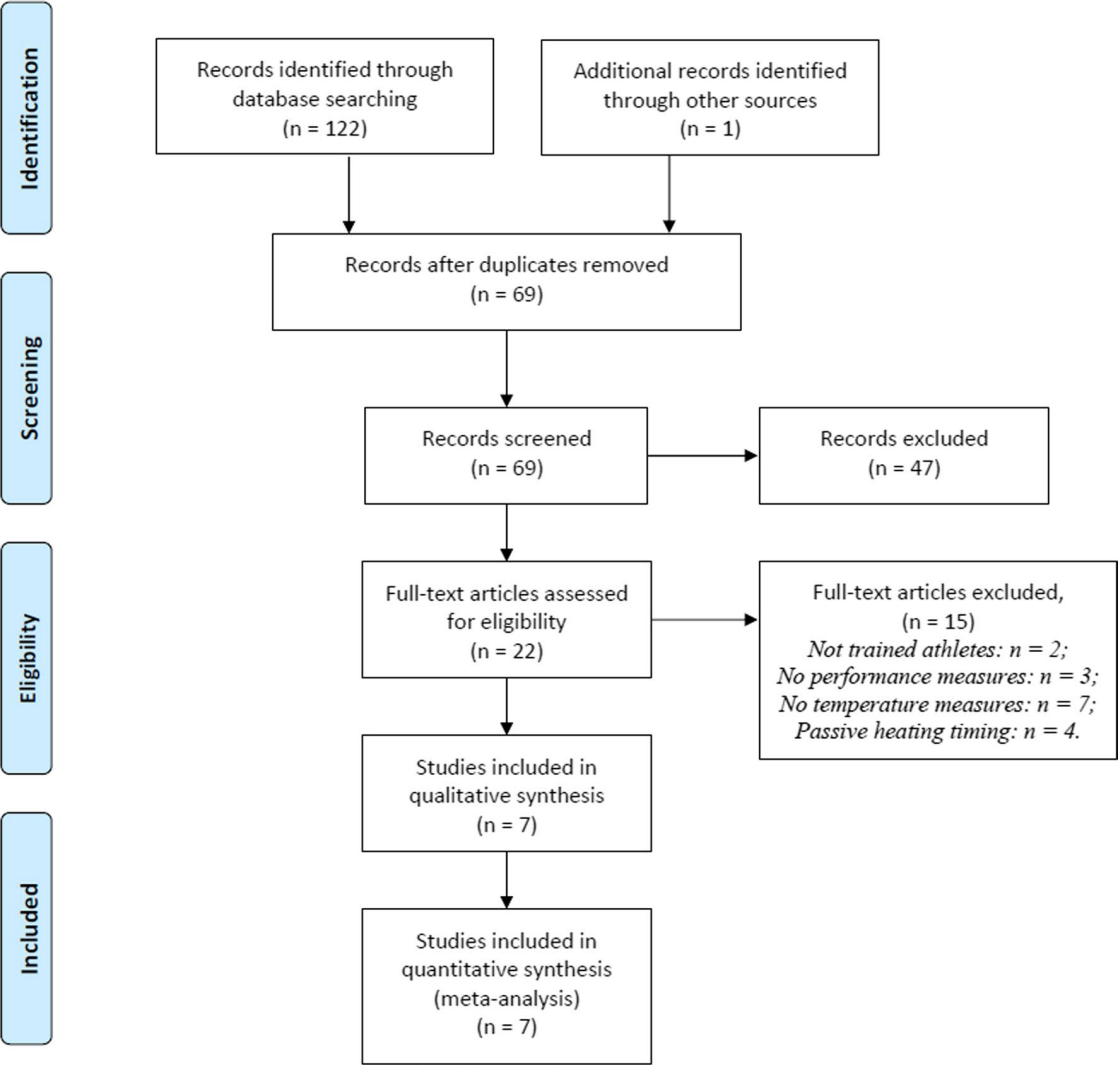
PRISMA (Preferred Reporting Items for Systematic Reviews and Meta-Analysis) study flow diagram

The initial search identified 123 articles with potential relevance. After removing duplicates and studies that were not specific to passive warm-up strategies, a manual screening according to the abstract was performed, and those that were not relevant were excluded. Two authors (G.C., M.B.) independently assessed articles against the inclusion criteria. From these studies, 22 original full-text research articles were assessed for eligibility, and those that did not meet the inclusion criteria were excluded. For the quantitative analysis, seven articles were included (Fig. 1).

Once the articles were removed based on the exclusion criteria, the final papers underwent an assessment of quality, using a modified version of the Physiotherapy Evidence Database (PEDro) scale. Due to the specificity of the topic, the scale was altered and questions 3–6 were replaced with more pertinent related questions. The PEDro scale is an 11-item scale designed to measure the methodological quality of experimental studies, with the reliability of the database being previously established [17]. Each satisfied criterion (except item 1, which pertains to external validity) is graded one point to the overall score (possible range of 0–10 points). Eight items on the scale assess the internal validity with the remaining two analysing the sufficiency of the statistical information. In agreement with previous reviews, studies with a score of 5 out of 10 or higher were included in the review to improve the credibility of the analysis [18, 19].

### Data extraction

From the included articles, data on sample size and participant sex, passive heating strategy and the duration of the passive heating strategy, physiological temperature changes during the intervention, exercise protocol and the performance outcomes were extracted. In addition to the ambient temperature and humidity. Outcome data were presented using a Microsoft Excel 2016 spreadsheet (Microsoft, Redmond, WA, USA) (Table 2).

**Table 2:** Physiological and performance changes in exercise performance following a passive heating intervention

Study	Participants	Conditions		Intervention		Phys. changes (°C)		Performance task	
		Temperature (°C)	Humidity (%)	Passive heating method	Duration (min)	Baseline—post rec	Post WU—post rec	Exercise protocol	Performance changes
Cook et al. [10]	3 Males 3 Females	20	70–75	Tracksuit Top (CON)	15	T_Tymp_ = ↓ 1.0		3 × 20 m Sledpull sprint (3 min recovery)	TTC ↓ 3.53% ± 0.61% for HEAT versus CON (*P* < 0.05)
				Blizzard Survival Garment (HEAT)		T_Tymp_ = ↓ 0.1			
Cowper et al. [14]	10 Males	8	50	Tracksuit Top (CON)	25		T_Tymp_ = ↓ 0.93	2000 M Rowing TT	TTC ↓ 1.1% for HEAT versus CON (*P* < 0.01)
				Tracksuit Top with integrated electric heating elements (HEAT)			T_Tymp_ = ↑ 0.54		
Falkner et al. [9]	11 Males	15.9 ± 0.3	54.0 ± 4.0	Tracksuit (CON)	30		T_Rec_ = ↓ 0.2	30 s maximal sprint cycle	PPO ↑ 9.1% HEAT versus CON (*P* < 0.05) PPO ↑ 4.1% HEAT versus INS Mean PO ↑ 7.8% HEAT versus CON Mean PO ↑ 8.4% HEAT versus INS
				External heated pants (HEAT)			T_Rec_ = ↓ 0.3		
				Insulated tracksuit pants (INS)			T_Rec_ = ↓ 0.1		
Falkner et al. [20]	10 Males	16.1 ± 0.2	53 ± 2.0	Tracksuit (CON)	30		T_Rec_ = ↓ 0.1 T_Musc_ = ↓ 1.5	30 s maximal sprint cycle	PPO ↑ 11.1% HEAT versus CON (*P* < 0.005) PPO ↑ 1.2% HEAT versus HEATHEAT Mean PO ↑ 4.3% HEAT versus CON (*P* < 0.005) Mean PO ↑ 0.7% HEAT versus HEATHEAT
							T_Rec_ = ↓ 0.3 T_Musc_ = ↓ 1.3		
				External heated pants during REC (HEAT)			T_Rec_ = ↓ 0.1 T_Musc_ = ↓ 1.2		
				External heated pants during WU and REC (HEATHEAT)					
Killduff et al. [21]	20 Males	19.5 ± 0.3	63.0 ± 3.0	Tracksuit (CON)	15		T_Pill_ = ↓ 0.56	CMJ and 6 × 40 m (20 + 20 m) shuttle sprints separated by 20 s of passive recovery (RST)	CMJ PPO ↑ 3.5% HEAT versus CON (*P* < 0.001) RST Mean ↓ 0.7% HEAT versus CON (*P* < 0.05)
				Blizzard survival garment (HEAT)			T_Pill_ = ↓ 0.19		
West et al. [5]	16 Males	21.4 ± 0.4	61.0 ± 2.0	Tracksuit (CON)	20		T_Pill_ = ↓ 0.64	CMJ and 6 × 40 m (20 + 20 m) shuttle sprints separated by 20 s of passive recovery (RST)	CMJ PPO ↑ 3.2% HEAT versus CON (*P* < 0.05) RST Mean ↓ 4% HEAT versus CON (*P* < 0.05)
				Blizzard survival garment (HEAT)			T_Pill_ = ↓ 0.2		
Wilkins and Havenith [13]	8 Males	23.4 ± 0.1	55.8 ± 1.4%	Tracksuit (CON)	30	T_Tymp_ = ↓ 0.8		Max plyometric press-ups (PPU) and 50 m freestyle swimming time trial	PPU Peak Force ↑ 10.1% HEAT versus CON (*P* < 0.05) TTC ↓ 0.83% for HEAT versus CON (*P* = 0.06)
	4 Females			External heated jacket (HEAT)		T_Tymp_ = ↓ 0.8			

CMJ, Countermovement jump; REC, Recovery; RST, Repeated sprint time; PO, Power output; PPO, Peak power output; PPU, Plyometric press-up; T_Tymp_, Tympanic temperature; T_Rec_, Rectal thermometry; T_Pill_, Ingestible core sensor; T_musc_, Muscle temperature; TT, Time trial; TTC, Time to completion; WU, Warm-up; ↑, Increase; ↓, Decrease

### Meta-analysis

To analyse the impact of passive heating strategies on exercise performance, the mean and standard deviation of performance outcomes and physiological effects was inputted in a Microsoft Excel 2016 spreadsheet (Microsoft, Redmond, WA, USA). Cohen’s d was used to calculate effect sizes (ES), this was categorised as follows: < 0.2 = trivial effect size, 0.2–0.5 = small effect size, 0.5–0.8 = medium effect size and > 0.8 = large effect size [22]. Following this, the standard error of the effect size was calculated.

Meta-analyses and forest plots were produced using commercially available software (JASP Team, Amsterdam, Netherlands). The Cochrane’s Q test was performed to test the null hypothesis of heterogeneity to identify whether studies had similar effect sizes. Statistical heterogeneity was defined as I^2^ small (< 30%) and large (> 50%) respectively [23]. If specific data was not presented in the study, the corresponding author was contacted and given 30 days to respond. If no response was received, the data was excluded from the meta-analyses.

## Results

### Study identification and selection

Initially, a total of 123 studies were retrieved from the literature search. From these 123 articles, duplicates were removed (n = 54). The titles and abstracts of each entry (69 articles) were then screened for their relevance, which resulted in the rejection of 47 articles from the analysis. Following this trimming, the full texts of the remaining 22 articles were reviewed. Of the 22 articles, 15 were excluded due to their irrelevance to the topic area. After one article was retrieved and accepted through other source, seven articles were accepted for the systematic review completing the full screening process. A summary of the process involved in retrieving suitable studies can be viewed in the flowchart presented based on the process developed for the quality of reporting of meta-analyses [15] (see Fig. 1).

### Methodological quality

After the application of the pre-defined exclusion criteria, the remaining full-text articles were assessed for methodological quality via a Modified Physiotherapy Evidence Database (PEDro) scale. All seven of the remaining articles achieved a PEDro score of 5 or above and thus were included in the review commentary. The mean range of quality scores was 7.6 ± 1.1 (range, 6–9 out of 10) (Additional file 1: Table S1).

### Study characteristics

A chronological analysis of the articles that comprised this review showed a recent interest in this area of research, with all the included studies published in the last eight years (2013–2021). In the seven eligible articles, outcomes were presented for 85 participants (individual study sample sizes ranging from six to 20 participants), which were well-trained athletes of whom 78 were male and seven were female. Exercise modalities included cycling, rowing, bobsleigh, repeated sprints, plyometrics and bodyweight exercises. Considerable methodological variations also existed with regards to the active warm-up and strategies of passive heating. The most prominent variations were in the passive heating duration and choice of garment (see Table 2).

### Performance responses to passive heating

**Fig. 2:**
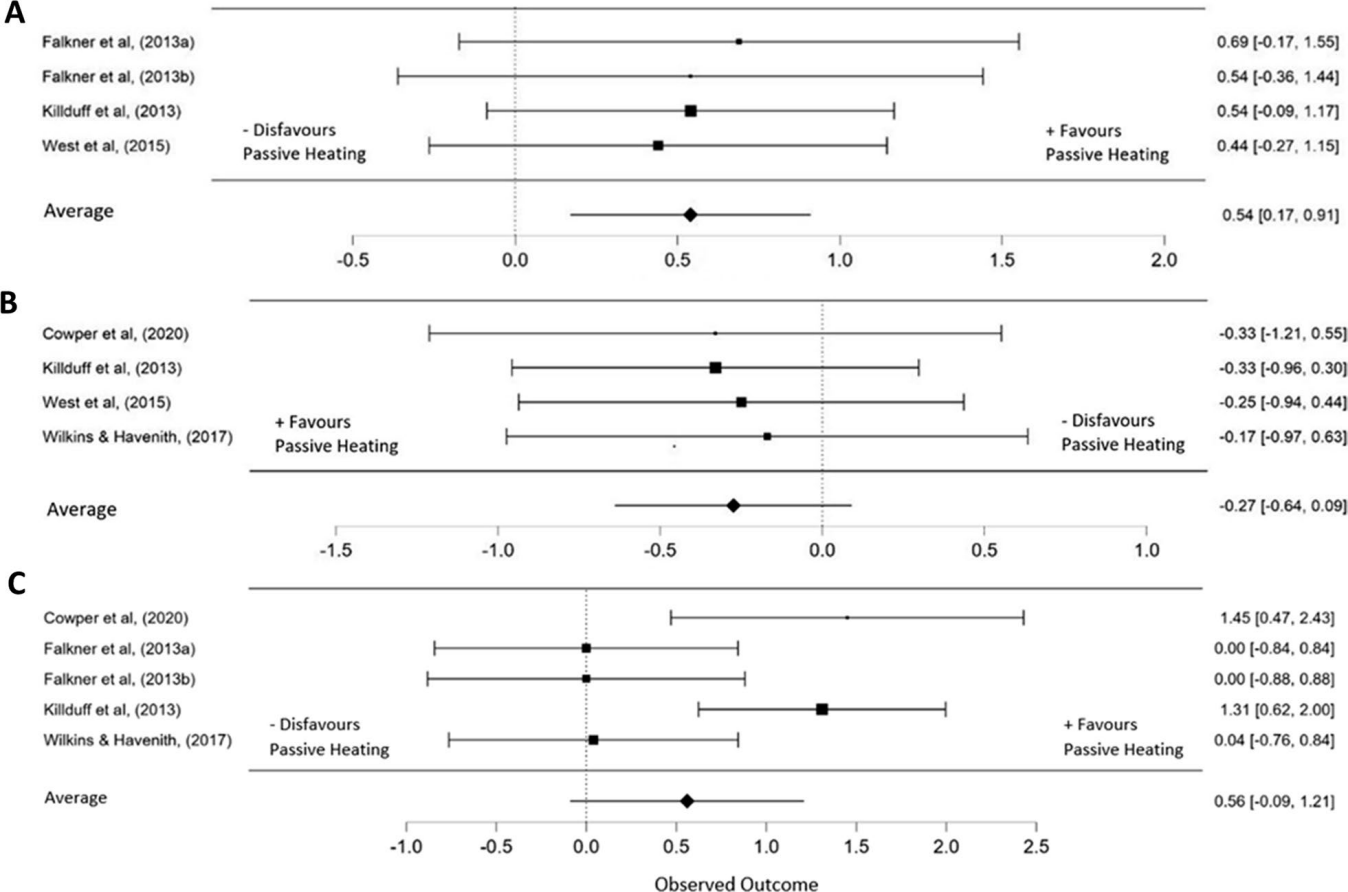
Forest Plot of multilevel meta-analysis comparing **A** peak power output **B** time to completion activities **C** core temperature, post passive heating jacket invention following an active warm-up. The study-specific intervals represent individual effect size estimates and sampling error. The black squares represent the pooled estimate generated with inference along with a 95% confidence interval

All seven studies found a significant improvement (*P* < 0.05) in one or more performance parameters following the passive heating intervention. Four out of the seven eligible studies assessed peak power output (PPO) which was measured in the form of cycling [9, 20], and countermovement jumps [5, 21]. The overall effect size suggests a significant improvement in PPO following a passive heating strategy (ES 0.54 [95% CI 0.17 to 0.91], *P* = 0.005, I^2^ = 0%). Additionally, four out of seven studies measured performance in a form of time trial, which included rowing [14], repeated sprints [5, 21] and swimming [13]. Interestingly, the improved PPO did not translate into an overall effect for time trial performance (ES =  − 0.27 [95% CI − 0.64 to 0.09], *P* = 0.141, I^2^ = 0%) (Fig. 2).

### Temperature responses to passive heating

Following the passive heating period between the active warm-up and performance, five out of the seven included studies reported core temperature readings. Three out of the five studies implemented aural tympanic thermometry [10, 13, 14], two implemented rectal thermometry [9, 20], and two implemented ingestible sensors to measure core temperature [5, 21]. Overall, no significant effect was found in core temperature following the application of a heated garment (ES = 0.56 [95% CI − 0.09 to 1.21, *P* = 0.09, I^2^ = 68%) (Fig. 2). Two out of the seven studies implemented muscle temperature measurements from depths of 1, 2 and 3 cm in the vastus lateralis. Faulkner and colleagues found muscle temperature to be approximately 1 °C higher at a depth of 1 cm (*P* < 0.001) and 0.4 °C higher at 3 cm (*P* < 0.01) following the use of heated trousers against standardised tracksuit bottoms [9]. Furthermore, in a supplementary study, Faulkner and colleagues observed that following the passive rest period, muscle temperature declined in all conditions, however, remained higher during the use of heated trousers at all muscle depths compared to standardised tracksuit pants and insulated pants (*P* < 0.001) [20].

## Discussion

This study has identified that completing a passive warming strategy with the use of heated garments between an active warm-up and competition can improve subsequent performance in the form of increased PPO (ES = 0.54 [95% CI 0.17 to 0.91]. From the studies included, improvements have been demonstrated in aerobic and anaerobic type sports such as rowing [14], cycling [9, 20] and swimming [13]. Improvements have also been shown in the activities of vertical jumping [5, 21] and repeated sprint performance [5, 10, 21]. The included literature suggests that maintaining an elevated muscle temperature is only beneficial in the early stages of exercise, however, it should be noted that having a much greater start in a time trial, does not necessarily mean subsequent exercise performance will be improved due to elevated afferent feedback [24]. However, despite a consensus on the effect of heated garments, the degree of improvement varied widely, from less than 1 to over an 11% improvement, however, the studies included have exhibited sufficient quality as evidenced through extensive critique. The use of multiple variations of active and passive warm-up protocols, the wide variation in results may be attributable to the specifics of the warm-up practices employed, post-warm-up, transition period strategies and the exercise itself. This emphasises the need for continued research to determine which methods of warm-up are best for a given sport or activity.

### Passive warm-up strategies and exercise performance

Asmussen and Boje [25] and Bergh and Ekblom [26] reported that the velocity-dependent effect of muscle temperature on maximum external power to be ~ 4% higher in force and power per 1 °C rise in quadriceps muscle temperature. Furthermore, per 1 °C increase in muscle temperature, observed a 2–5% [27], and a 2–10% [28] increase in peak power during exercise performance.

A passive warming strategy, unlike a continuous active warm-up, during a transition period between an active warm-up and exercise performance allows a rise in muscle and/or core temperature without the reduction of energetic substrates [9, 20]. Initial studies emphasising passive warming strategies were solely laboratory based, with the method of increasing body temperature being accomplished by methods, such as hot baths/showers. Although impactful, these passive warm-up strategies are not often practical in a sporting scenario. Therefore, alternate methodologies of passive warming have been sought after, given that: (1) a lengthy period is expected (transition phase), between the end of the warm-up and the beginning of an event; (2) muscle temperature starts to fall immediately following exercise termination; and (3) substantial declines in body temperature occurs as early as ~ 15–25 min post-exercise [4, 5].

### External heating garments and blizzard survival jackets and performance

All of the included studies in this review used either blizzard survival jackets or external heating garments, the majority of heated garments used across various sporting activities have heat filaments in the fabric. Faulkner et al. [9] reported an improvement in muscle temperature (1 °C rise in muscle temperature at a depth of 1 cm and a 0.4 °C rise at a 3 cm depth) and ~ 9% improvement in relative and PPO during a sprint cycling task when using an active warm-up and heated tracksuit pants in the 30-min transition period in comparison to standard tracksuit pants [9]. Additionally, further research by Faulkner and colleagues reported that muscle temperature remained increased when wearing heated tracksuit pants solely during the transition period (36.9 ± 0.3 °C) and when worn throughout the active warm-up and transition phase (37.0 ± 0.2 °C) in contrast to only an active warm-up (36.6 ± 0.3 °C) [20]. Although, an additional performance benefit was not found when wearing the heated tracksuit pants during an active warm-up as well as during the transition phase [20]. Supporting this, Cook et al. reported that wearing a blizzard survival jacket produces a rise in tympanic temperature and improved a 20 m sled sprint performance [10]. Additionally, Kilduff et al. reported an improved repeated sprint performance in elite rugby players when an active warm-up was followed by wearing a blizzard survival jacket throughout a 15-min transition period [21]. The decrease in core temperature during the transition phase was minimised when the blizzard survival jackets were worn (− 0.19 ± 0.08 °C) compared to the standardised tracksuit top (− 0.55 ± 0.10 °C). Therefore, participants began the tests with an elevated core temperature, suggesting that an increased core temperature prior to exercise can improve exercise performance [21].

The use of passive warming strategies are not common practice, however, the application of maintaining body and muscle temperature during a transition period is gaining recognition. Passive heat maintenance through the wearing of blizzard survival jackets and athletic heating garments appears to be an optimal technique in offsetting the reduction in core and/or muscle temperature and therefore improving exercise performance. However, athletic heating garments can have their limitations. Wired heated garments, do not provide uniform heat across the heating elements. Furthermore, for optimal heat transfer and increase in muscle temperature, the heating elements should be in direct contact with the skin, which involves the garment being tight to the skin [29]. Additionally, Faulkner et al. reported a significant decrease in muscle temperature during the inactivity period when the heated garments were worn, however, the decline in muscle temperature was significantly less compared to the application of standard tracksuit bottoms [9]. Furthermore, Raccuglia and colleagues demonstrated by using water-perfused heated trousers, heated to a higher temperature, 43 °C, successfully maintained and even increased muscle temperature in the passive recovery period following an active warm-up [30]. However, water perfused trousers are not very practical for use in a competition setting as they need to be connected to a heating system, consisting of a temperature-controlled water bath and powered water pump. Alternatively, numerous studies have reported a significant improvement in exercise performance following the application of battery-powered athletic heating garments which use integrated flexible heating elements [9, 13, 14, 20]. This allows consumers to use the garments portably, however without multiple batteries, for a limited time period.

All the reviewed literature found a significant improvement in exercise performance (*P* < 0.05); particularly time trial performances, which have shown to display a wide variety of percentage increases. When examining these in more detail, the type of exercises might influence the overall percentage improvement. For example, Cowper et al. exhibited a performance increase of 1.1%, this slight increase in performance, might be because of the competition duration being long (> 5 min) in comparison to the other studies which are all predominantly anaerobic and short-lasting in nature [14]. Limited studies have determined the physiological outcomes of passive warming for long-duration performances. This might be because a well-known limiting physiological factor for long-duration performances is excessive bodily heat [31, 32].

A rise in core temperature before exercise might be detrimental to long duration performance due to impaired thermoregulatory mechanisms [33, 34] and/or a decrease in heat storage capacity [35]. This increase in body temperature, may create a greater dependence on evaporative heat loss, thus an increase of sweating. Therefore, an individual may incur sub-optimal hydration over a prolonged period and subsequently hinder exercise performance [36]. Furthermore, González-Alonso et al. [33] found a detriment in time till exhaustion performance in hot environmental conditions (40 °C) when comparing 36 (CON), and 40 °C (HEAT) pre-exercise water immersions for 30 min. Observing that a reduction in performance was attributed to increases in heart rate and reductions in stroke volume paralleled the rise in core temperature [33].

When a heated jacket is utilised following an active warm-up in cool environments, body temperature would be comparatively lower than if the same protocol was applied in standard ambient temperatures (18–20 °C) [37]. During colder environments, a delayed duration that the body takes to reach critical core temperature would occur and performance might improve. Alternatively, in standard ambient conditions, the use of a heated jacket may elevate the core temperature to critical levels and possibly decrease the capacity for exercise performance [14]. Due to the paucity of work in this area, the papers included in this systematic review have differing ambient temperatures. It is known that a change in ambient temperature can significantly alter the thermoregulatory profile at rest [38] and affect the ability to exercise [39]. As such, the ambient temperature is an important factor to consider when using interventions during the passive warming period. Therefore, further research is needed to determine the effect on performance following the use of passive heating garments in below ambient temperatures.

### Limitations

Several methodological problems in the studies reviewed could have impacted the outcomes reported. Some of the studies did not measure temperature change post active warm-up, therefore it might be difficult to distinguish whether the change in bodily temperature was due to the active warm-up or the passive heating garment. Furthermore, chosen studies in this systematic review and meta-analysis displayed small sample sizes, this led to many of the confidence intervals crossing the ‘‘zero point’’. While the conclusions from this review are based on mean data, it is important to state that even though none of the included studies displayed an absolute improvement in performance for passive heating when examining both the means and confidence intervals, no studies showed a mean or absolute decrease in performance. This demonstrates that passive heating during the period between active warm-up and performance has no effect on performance. There are certain weaknesses in this review, relatively small number of participants in some of the studies which increases the potential effects of chance. Furthermore, the limited number of studies included in the systematic review and meta-analysis and the studies that were included, some authors were unable to provide raw data to fully complete analyses.

The findings of this systematic review and meta-analysis offer a partial but best available evaluation of the influence of passive heating techniques prior to sport and exercise performance. This review aimed to eliminate possible sources of bias by employing a systematic review method, however, this does not ensure the absence of bias. Furthermore, a modified version of the PEDro scale was applied to distinguish between the quality of different studies. The modified PEDro scale has not likely to have biased our decisions since points are only granted to studies when the criteria are clearly fulfilled. Furthermore, after a precise reading of the included research articles, if it was not evident that the criterion was reported, a point was not presented for that specific criterion.

## Future considerations

Further research is necessary to determine the optimum procedure for passive warm-up strategies, including the length of time to wear the garments, garment temperature and the placement of the heating elements embodied into the garment. Furthermore, research on whether passive heating strategies could be applied to scenarios where it is difficult to maintain core temperature from metabolic heat production alone, such as repeated-sprint sports which are separated by low to moderate activity. Specifically, in below ambient conditions, where the decline in core and muscle temperature is excessive throughout a lengthy transition.

## Conclusion

Passive heating devices have been shown to reduce the decline in muscle temperature, and therefore have been found to enhance PPO during exercise. Additionally, a favourable yet not significant reduction in the decline of core temperature was observed. Large heterogeneity, low participant numbers and a range of methodologies, shows a necessary scope of additional research to observe the effects of core and muscle temperature.

## Supplementary Information

**Additional file 1. Table S1.** Methodological quality scores of the included studies.

**Additional file 2.** Raw data of meta-analysis.

## Data Availability

All data generated or analysed during this study are included in this published article (and its Additional files 1 and 2).

## Abbreviations

CMJ: Countermovement jump
ES: Effect size
PEDro: Physiotherapy evidence database
PO: Power output
PPO: Peak power output
PPU: Plyometric press-up
REC: Recovery
RST: Repeated sprint time
T_Pill_: Ingestible core sensor
T_Rec_: Rectal temperature
T_Tymp_: Tympanic temperature
TTC: Time to completion
WU: Warm-up

## References

[1] Park HK (2018). The effect of warm-ups with stretching on the isokinetic moments of collegiate men. J Exerc Rehabil.

[2] Silva LM (2018). Effects of warm-up, post-warm-up, and re-warm-up strategies on explosive efforts in team sports: a systematic review. Sports Med.

[3] Bishop D (2003). Warm up I—potential mechanisms and the effects of passive warm up on exercise performance. Sports Med.

[4] Mohr M (2004). Muscle temperature and sprint performance during soccer matches—beneficial effect of re-warm-up at half-time. Scand J Med Sci Sports.

[5] West DJ (2013). Influence of post-warm-up recovery time on swim performance in international swimmers. J Sci Med Sport.

[6] Zochowski T, Johnson E, Sleivert GG (2007). Effects of varying post-warm-up recovery time on 200-m time-trial swim performance. Int J Sports Physiol Perform.

[7] Carlile F (1956). Effect of preliminary passive warming on swimming performance. Res Q Am Assoc Health Phys Educ Recreat.

[8] Muido L (1946). The influence of body temperature on performances in swimming. Acta Physiol Scand.

[9] Faulkner SH (2013). Reducing muscle temperature drop after warm-up improves sprint cycling performance. Med Sci Sports Exerc.

[10] Cook C (2013). Designing a warm-up protocol for elite bob-skeleton athletes. Int J Sports Physiol Perform.

[11] Russell M (2015). A passive heat maintenance strategy implemented during a simulated half-time improves lower body power output and repeated sprint ability in professional rugby union players. PLoS ONE.

[12] McGowan CJ (2016). Heated jackets and dryland-based activation exercises used as additional warm-ups during transition enhance sprint swimming performance. J Sci Med Sport.

[13] Wilkins EL, Havenith G (2017). External heating garments used post-warm-up improve upper body power and elite sprint swimming performance. Proc Inst Mech Eng Part P J Sports Eng Technol.

[14] Cowper G, Barwood M, Goodall S (2021). Improved 2000-m rowing performance in a cool environment with an external heating garment. Int J Sports Physiol Perform.

[15] Moher D (2015). Preferred reporting items for systematic review and meta-analysis protocols (PRISMA-P) 2015 statement. Syst Rev.

[16] Methley AM (2014). PICO, PICOS and SPIDER: a comparison study of specificity and sensitivity in three search tools for qualitative systematic reviews. BMC Health Serv Res.

[17] Maher CG (2003). Reliability of the PEDro scale for rating quality of randomized controlled trials. Phys Ther.

[18] Russell M, Kingsley M (2014). The efficacy of acute nutritional interventions on soccer skill performance. Sports Med.

[19] Kromer TO (2009). Effects of physiotherapy in patients with shoulder impingement syndrome: a systematic review of the literature. J Rehabil Med.

[20] Faulkner SH (2013). External muscle heating during warm-up does not provide added performance benefit above external heating in the recovery period alone. Eur J Appl Physiol.

[21] Kilduff LP (2013). The influence of passive heat maintenance on lower body power output and repeated sprint performance in professional rugby league players. J Sci Med Sport.

[22] Cohen J (1992). A power primer. Psychol Bull.

[23] Higgins JPT, Thompson SG (2002). Quantifying heterogeneity in a meta-analysis. Stat Med.

[24] Amann M (2012). Significance of Group III and IV muscle afferents for the endurance exercising human. Clin Exp Pharmacol Physiol.

[25] Asmussen E, Bøje O (1945). Body temperature and capacity for work. Acta Physiol Scand.

[26] Bergh U, Ekblom B (1979). Influence of muscle temperature on maximal muscle strength and power output in human skeletal muscles. Acta Physiol Scand.

[27] Racinais S, Oksa J (2010). Temperature and neuromuscular function. Scand J Med Sci Sports.

[28] Sargeant AJ (1987). Effect of muscle temperature on leg extension force and short-term power output in humans. Eur J Appl Physiol.

[29] Wang FM (2010). A review of technology of personal heating garments. Int J Occup Saf Ergon.

[30] Raccuglia M (2016). Post-warm-up muscle temperature maintenance: blood flow contribution and external heating optimisation. Eur J Appl Physiol.

[31] Kozlowski S (1985). Exercise hyperthermia as a factor limiting physical performance—temperature effect on muscle metabolism. J Appl Physiol.

[32] Romer LM, Barrington JP, Jeukendrup AE (2001). Effects of oral creatine supplementation on high intensity, intermittent exercise performance in competitive squash players. Int J Sports Med.

[33] Gonzalez-Alonso J (1999). Influence of body temperature on the development of fatigue during prolonged exercise in the heat. J Appl Physiol.

[34] Fortney SM (1984). Effect of hyperosmolality on control of blood-flow and sweating. J Appl Physiol.

[35] Nadel ER (1987). Prolonged exercise and high and low ambient temperatures. Can J Sport Sci.

[36] Sawka MN (1998). Hydration effects on temperature regulation. Int J Sports Med.

[37] Marino FE (2002). Methods, advantages, and limitations of body cooling for exercise performance. Br J Sports Med.

[38] Wagner JA, Horvath SM (1985). Influences of age and gender on human thermoregulatory responses to cold exposures. J Appl Physiol.

[39] Galloway SD, Maughan RJ (1997). Effects of ambient temperature on the capacity to perform prolonged cycle exercise in man. Med Sci Sports Exerc.

